# Dissociation in cross-feature integration between behavioral and pupil dilation responses in auditory deviant detection

**DOI:** 10.1016/j.isci.2026.116013

**Published:** 2026-05-20

**Authors:** Nahaleh Fatemi, Hsin-I Liao, Mounya Elhilali

**Affiliations:** 1 Electrical and Computer Engineering, Johns Hopkins University, Baltimore, MD, USA; 2 NTT Communication Science Laboratories, NTT, Inc., Atsugi, Japan

**Keywords:** Neuroscience, Behavioral neuroscience, Sensory neuroscience

## Abstract

The detection of deviant events in complex soundscapes requires tracking fluctuations across multiple acoustic features such as pitch, timbre, and loudness. While both behavioral measures and pupil dilation responses (PDR) are sensitive to individual feature changes, it remains unclear how these cues are integrated in complex acoustic scenes. We examined cross-feature interactions underlying auditory deviance using a deviant-detection task and PDR recordings as participants listened to dynamic soundscapes with deviant events. Using the Dynamic Regularity Extraction (D-REX) model, we quantified feature-specific and integrated surprisal to relate stimulus structure to observed responses. Constrained regression analyses showed that behavioral responses exhibited clear cross-feature integration, whereas PDRs were better explained by feature-specific surprisal with limited evidence of cross-feature interactions. These findings highlight a dissociation between physiological and behavioral responses and provide a framework for comparing how different response modalities relate to the acoustic structure of auditory scenes.

## Highlights

• Behavioral responses reflect integration across pitch, timbre, and loudness
• Pupil dilation reflects feature-specific surprisal with limited interaction
• D-REX links stimulus surprisal to behavioral and physiological responses
• Behavioral and pupil responses dissociate in cross-feature integration

## Graphical abstract

**Figure:**
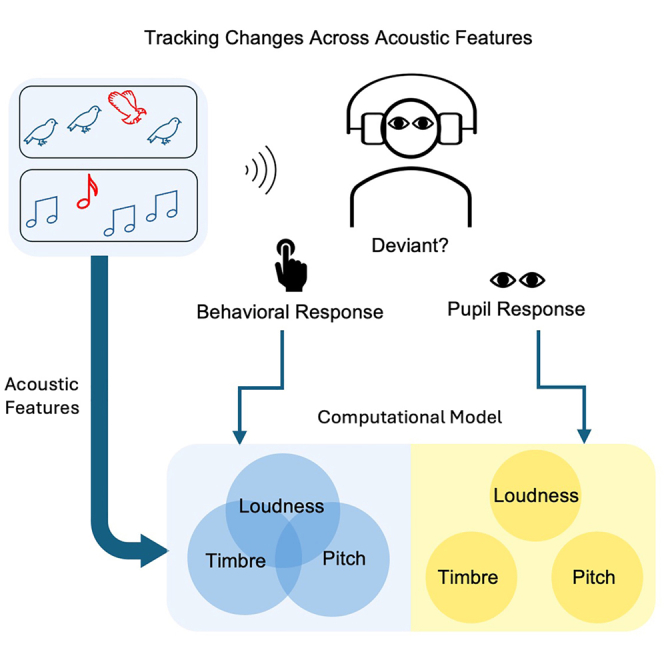

## Introduction

Auditory deviance refers to mismatches with expected sound patterns within complex acoustic scenes and plays an important role in the perceptual organization of these environments.^1^ Sudden changes in pitch, loudness, or timbre can signal important or unexpected events, yet the brain’s computation of deviance is not driven by isolated features alone. Instead, it reflects a dynamic integration of multiple acoustic dimensions in the auditory system. Despite growing interest in auditory deviant detection, it remains unclear how physiological signals like pupil dilation relate to behavioral responses in the context of multidimensional acoustic changes.

Pupillary dilation offers a non-invasive physiological measure associated with responses to unexpected sounds, reflecting arousal mediated in the midbrain, including locus coeruleus (LC) and superior colliculus.^2^ It has been shown to serve as a reliable index of arousal and cognitive effort and to reflect levels of attention.^3^^,^^4^ Prior research shows that deviant sounds, such as those with greater loudness, elicit larger pupil dilations, likely reflecting increased cognitive and sensory processing demands.^5^ This phasic pupil dilation response (PDR) is closely linked to LC activity, a key midbrain mechanism that is central for arousal and attention regulation.^6^ The LC also interacts with cortical regions that can modulate pupil responses via subcortical pathways.^7^ However, it remains unclear whether PDR is shaped by the integrative processes in cortical auditory processing or reflects sensitivity to deviant sounds without substantial contributions from interactions across acoustic dimensions.

Importantly, auditory deviance itself is inherently multidimensional. It is not determined by a single feature such as loudness, but rather by the interaction of multiple acoustic dimensions, including pitch, timbre, temporal structure, and statistical predictability. Prior studies provide clear evidence of such feature interactions. For instance, Bizley et al. demonstrated non-linear interactions between pitch and timbre representations in the primary auditory cortex of ferrets, suggesting that these features are not processed independently even at early cortical stages.^8^ Similarly, human psychophysical work by Allen and Oxenham revealed symmetric interference effects between pitch and timbre judgments, showing that variation in one feature dimension can perceptually mask or distort variation in another.^9^ These findings emphasize that the brain’s assessment of deviance likely involves more than additive contributions of individual acoustic features; rather, deviance arises from complex, potentially non-linear, feature integration processes.

In light of this complexity, it remains unclear how well physiological measures such as PDR reflect this multidimensional integration process and how such responses compare to behavioral measures of auditory deviant detection. Specifically, do PDR and behavioral performance capture similar patterns of feature interactions or do they reflect different sensitivities to particular acoustic dimensions? Addressing these questions can reveal whether PDR primarily reflects sensitivity to deviant stimuli or whether it is also influenced by interactions across acoustic dimensions similar to those observed in behavioral responses.

In the present study, we extend the investigation of PDR by exploring its sensitivity to deviant events embedded within auditory sequences composed of multiple, interacting acoustic features. We aim to determine whether PDR reliably reflects changes in auditory deviance across these different dimensions and whether its pattern of sensitivity aligns with that observed in behavioral performance. By comparing trends in PDR and behavioral data, we seek to identify whether these two response modalities show consistent or divergent patterns of feature integration, and how they differ in their relationship to acoustic feature structure.

To model and quantify these cross-dimensional feature interactions, we employ the Dynamic Regularity Extraction (D-REX) model, a computational framework designed to capture predictive processing in multi-feature acoustic sequences.^10^ This framework integrates information across different acoustic dimensions to compute feature-wise surprisal as a measure of statistical deviation from expected patterns, providing an approach inspired by predictive coding principles. Importantly, the D-REX model is fitted to both behavioral responses and PDR, enabling direct comparison of feature relationships across the two response modalities. By manipulating the model’s input parameters and examining its predictions, we can assess how well the statistical structure of the acoustic environment accounts for variance in both physiological and behavioral outcomes. This approach offers a principled method for probing computational accounts of auditory deviant detection through the lens of both behavioral and physiological responses.

**Figure 1:**
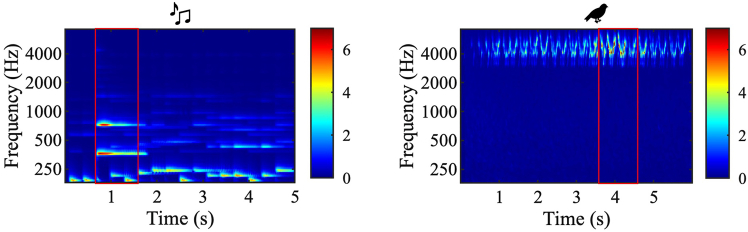
Examples of the stimuli Auditory spectrograms are shown for an example stimulus from the Music experiment (left) and the Nature experiment (right). The red box indicates the deviant token.

**Figure 2:**
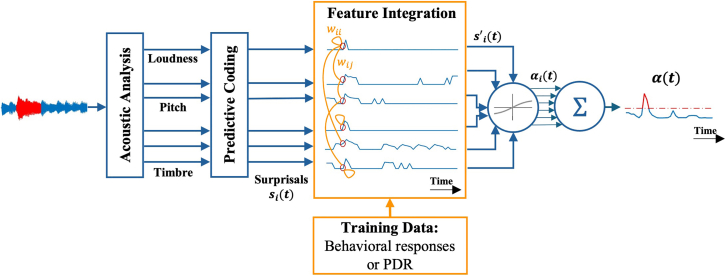
Overall flow of the model The sound wave is used to extract six acoustic features, each representing an acoustic dimension. The resulting features are passed through a Bayesian tracking model (D-REX) to compute the surprisals. The surprisals are used as input to a regression model, which can be trained on behavioral responses or PDR. Finally, the regression model will output the probability of a deviant event at each time point.

Building on our goal to distinguish how conscious perception and autonomic arousal track auditory deviance, this study examines how multidimensional acoustic information is represented across behavioral and physiological responses. Participants performed a deviant-detection task in which they listened to short musical clips or birdsong recordings and identified when a target sound deviated from the background along one or more acoustic dimensions—loudness, pitch, or timbre (Figure 1). Each perceptual dimension was quantified using computational features that extracted the envelope, pitch, spectral content, as well as spectral and temporal modulations.^11^^,^^12^ For each acoustic feature, we first computed a time-varying surprisal signal using the D-REX model, which quantifies how unexpected the auditory input is over time. Surprisal here is strictly the model’s output and should not be confused with deviance. Nonetheless, surprisal provides a strong predictor of deviant events, capturing when and where unexpected changes occur in the auditory input. These feature-specific surprisals were analyzed independently to assess their individual contributions to behavioral and physiological responses. We then combined them using a logistic regression trained separately on behavioral and pupillary data to model how multiple acoustic dimensions interact to predict the occurrence of deviant tokens and reproduce each response type. The overall framework is illustrated in Figure 2. By comparing the learned feature interactions and predictive performance of the two models, we evaluate how behavioral and pupillary responses differ in representing the integration of acoustic information.

## Results

Behavioral responses to deviant sound tokens reveal interdependent integration of pitch, timbre, and loudness. Statistically significant cross-feature interactions are observed when analyzing participants’ behavioral responses (see STAR Methods). Within-subjects ANOVA results outlined in Table 1 show that pitch and loudness have a significant effect on participants’ responses in both the Music and Nature experiments; however, timbre significantly affects responses only in the Nature experiment. A significant interaction between pitch and loudness is also observed in the Nature experiment, indicating that variation in one feature influences perception of the other. Pitch and timbre show significant interactions in both experiments. These findings are in line with results obtained by Kaya and Elhilali.^13^

**Table 1:** Table showing feature interactions based on subject’s behavioral responses

Effects	Music F (*p*)	Nature F (*p*)
**P**	26.35(<0.001)	47.28(<0.001)
**L**	13.74(<0.01)	**66.07 (**<**0.001)**
Tb	0.06(0.81)	7.38(<0.02)
Tf	0.04(0.84)	11.30(<0.01)
P, L	0.29(0.60)	6.44(0.02)
P, Tb	19.90(<0.001)	4.42(0.05)
P, Tf	1.39(0.26)	1.16(0.30)
L, Tb	0.61(0.45)	2.49(0.14)
L, Tf	0.01(0.94)	0.08(0.78)
**Tb,Tf**	25.27(<0.001)	52.57(<0.001)
P, L, Tb	0.29(0.60)	1.40(0.26)
P, L, Tf	0.05(0.83)	1.29(0.28)
**P, Tb, Tf**	8.67(0.01)	21.83(<0.001)
L, Tb, Tf	4.26(0.06)	5.73(0.03)
P, L, Tb, Tf	0.65(0.43)	2.28(0.15)

Bolded values indicate statistical significance (*p* < 0.05). P, Pitch; L, Loudness; Tb, Timbre-background; Tf, Timbre-foreground.

**Figure 3:**
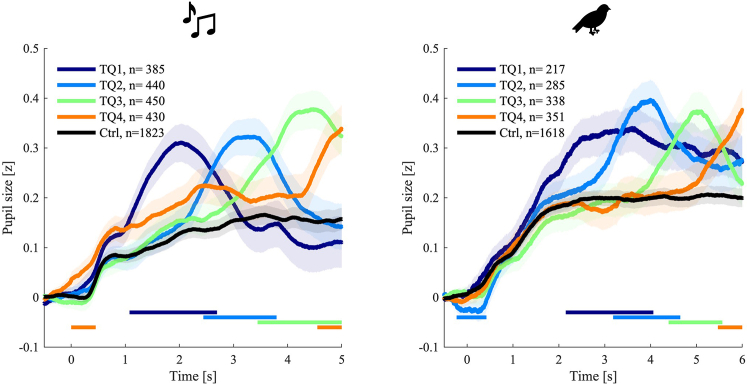
Average PDR for control trials and each time quadrant (Left) Music and (right) Nature experiments. *n* is the number of trials that were used in calculating the average PDR and the shaded area indicates the standard error of the mean (SEM). The horizontal lines indicate a significant difference between the non-control PDR (colored lines) and the control PDR (black line) (linear mixed-effects (LME) analyses: *t* value>2 for more than 200 consecutive samples).

To complement behavioral data, PDRs offer an independent physiological measure of auditory deviance. Analysis of participants’ PDR across time reveals a phasic trace following the deviant token. The analysis of participants’ PDR in different time quadrants throughout the trials shows a clear phasic PDR following the deviant stimuli with approximately 1-s delay. Figure 3 illustrates the average PDR traces across all trials and time segments for both Music and Nature stimuli, highlighting reliable physiological sensitivity to acoustic change. This suggests that PDR provides a time-locked index of auditory novelty.

**Figure 4:**
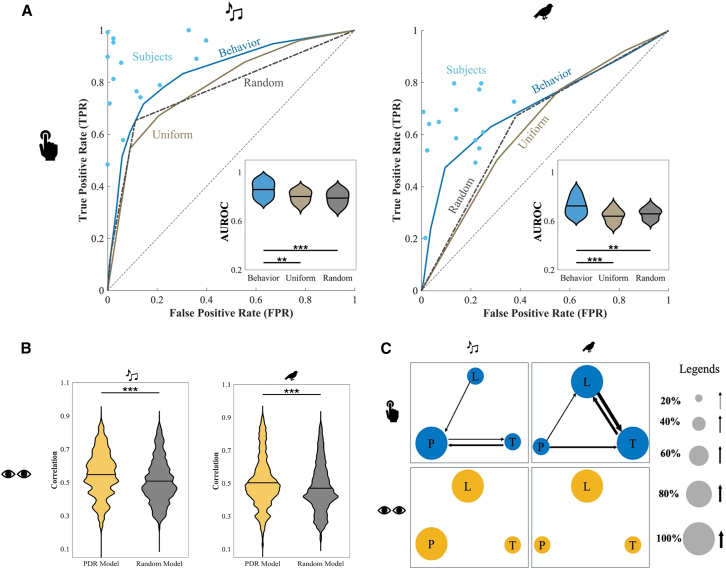
Behavioral and pupil model analysis and feature interaction weights (A) ROC and area under the ROC (AUROC) for the behavioral paradigm. The displayed ROC curve is obtained by aggregating data from all test subjects. Alternatively, an ROC curve can be generated for each individual subject. To illustrate the variability in performance across subjects and across models, we analyze the distribution of AUROC scores. The violin plots show the distribution of AUROC values for each model across subjects; the width of each violin reflects the data density, and the central marker indicates the mean (pairwise Student’s *t* tests with Bonferroni correction: ∗∗p<0.01,∗∗∗p<0.001). (B) Correlation for the PDR and random models. Yellow and gray show the correlation between the PDR and random models, respectively. The violin plots show the distribution of correlation values for each model across subjects; the width of each violin reflects the data density, and the central marker indicates the mean (Student’s *t* test: ∗∗∗p<0.001). (C) Feature interactions. This figure shows the feature interaction weights resulting from training the model on behavioral responses (top row) and PDR (bottom row) for the Music (left column) and Nature (right column) experiments. The behavioral weights represent the average across subjects for each experiment (for subject-wise interaction weights, see Figure S1), while the PDR weights are computed as the average across all possible combinations of 8 subjects from the full set of subjects. The radius of each circle shows the magnitude of that feature’s weight, and the thickness of the arrows shows the cross-feature weights (L, loudness; P, pitch; T, timbre). Legends are shown on the right.

To probe the mechanisms underlying these behavioral and physiological responses, we assess how each response modality is captured by the predictive model. The behavioral model performance tracks participants’ detection accuracy. As shown in Figure 4A, the behavioral model closely mirrors participant detection performance, with receiver operating characteristic (ROC) curves demonstrating high alignment for both the Music and Nature experiments. Area under the ROC (AUROC) scores confirm that the behavioral model significantly outperforms both the uniform-weight and random baseline models in both experimental conditions (Music experiment: one-way ANOVA: F=8.63,p=7e−4; post hoc tests: behavior-uniform: p=8.1e−3; behavior-random: p=9e−4; Nature experiment: one-way ANOVA: F=9.62,p=4e−4; post hoc tests: behavior-uniform: p=4e−4; behavior-random: p=7.3e−3). Post hoc tests reveal that removing feature-specific weights (uniform model) or shuffling deviant timing (random model) significantly reduces model accuracy, underscoring the importance of learned cross-feature interactions in accounting for behavioral responses.

In parallel, the PDR-trained model captures key aspects of the physiological response, showing a strong correlation with actual pupil responses. As shown in Figure 4B, cross-correlation analysis demonstrates that the PDR model significantly outperforms the randomly trained baseline (Student’s *t* test: Music and Nature experiments: p<2.2e−16). These findings indicate that the model effectively reproduces both timing and magnitude of the pupil-based responses to auditory deviants, indicating that PDR provides a reliable physiological measure associated with deviant events.

Looking closely at learned weights, it is clear that cross-feature interactions are more pronounced in the behavioral model than in PDR model. Figure 4C visualizes the interaction weights among loudness (L), pitch (P), and timbre (T) for both models, reflecting both the contribution of each feature (circle size) and the strength of the cross-feature coupling (arrow thickness). Behavioral model weights reveal robust cross-feature influences consistent with the ANOVA results from participants’ behavioral responses (Table 1). For subject-wise interaction weights, see Figure S1. In contrast, the PDR model (Figure 4C, bottom row) exhibits no significant cross-feature interactions, suggesting that PDR is better explained by feature-specific surprisal. Overall, these findings highlight a distinction between how acoustic features shape behavioral responses versus PDRs.

**Figure 5:**
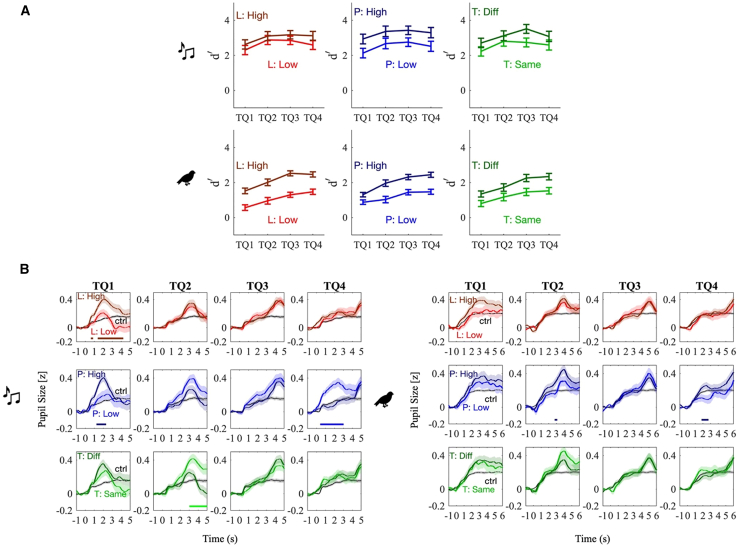
Behavioral performance and average PDR for different conditions and time quadrants (A) Participants’ d′ values (mean ± SEM) across time quadrants and trial conditions. Light-colored lines represent trials with a small change in the specified feature, while dark-colored lines indicate trials where the specified feature had a larger change. Different colors indicate change in the acoustic dimensions: L, loudness (red); P, pitch (blue); T, timbre (green). The High and Low legends show whether the change in the acoustic feature was large or small. (B) Average PDR in different conditions. (Left) Music and (right) Nature experiments. L indicates a change in loudness, P in pitch, and T in timbre. Darker lines show the PDR for larger change in the acoustic feature (high), and the lighter lines show a smaller change in the acoustic feature (Low). The horizontal lines indicate significant differences between the two colored lines (i.e., acoustic feature level differences) (LME analyses: t-value>2 for more than 200 consecutive samples). Dark lines indicate larger pupil sizes for the larger acoustic feature level difference than the smaller difference. Lighter lines indicate the opposite effect, i.e., larger pupil sizes for the smaller feature differences than the larger feature differences.

To investigate the temporal dynamics of auditory deviance, we examine how sensitivity to deviant events changes over time. Behavioral results reveal a gradual buildup in deviance detection sensitivity over the course of each trial. Participants’ $d^{\prime }$ (Figure 5A) shows significant improvement as the deviant token is presented later in the trial (F=8.739,p<0.001 for Music experiment; F=28.349,p<0.001 for Nature experiment). These results suggest that as listeners accumulate contextual information, their internal representations of the soundscape become more stable, enabling more efficient detection of unexpected events.

To assess whether similar dynamics is reflected in physiological responses, we examine pupil dilation across conditions and time. The average PDR for each condition (change in loudness, pitch, and timbre) is computed to examine how the responses change through time. The average PDR shows a significant difference along some dimensions and time quadrants, which are more visible in the Music experiment (Figure 5B). The difference in PDRs is most pronounced during the early time quadrants, suggesting that the PDR is consistent with an initial arousal response to the auditory scene. Over time, deviant stimuli elicit progressively weaker responses, consistent with reduced novelty. As the auditory stream continues, responses to deviant tokens may reflect additional processes beyond initial stimulus-driven responses, although their precise nature remains unclear.

**Figure 6:**
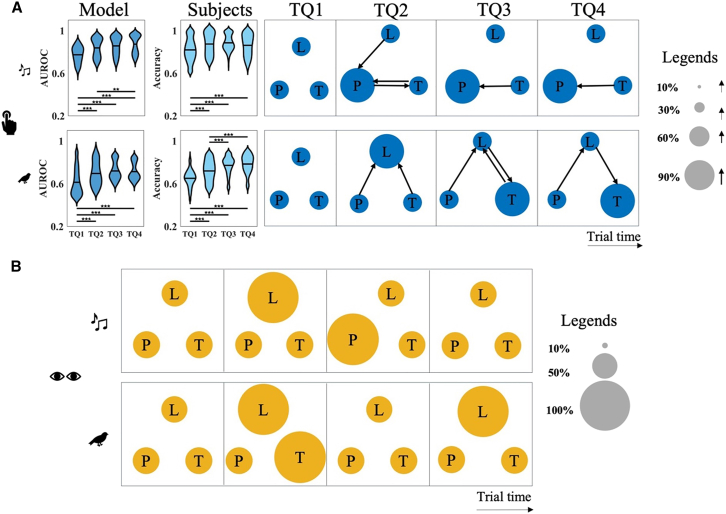
Time-dependent models by quadrants (A) Interaction weights and performances for the behavioral paradigm in each time quadrant (top row: Music experiment, bottom row: Nature experiment). The model AUROC and subjects’ accuracy is shown on the left for each time quadrant. The violin plots show the distribution of AUROC and accuracy values across participants; the width of each violin reflects the data density, and the central marker denotes the mean. The interaction weights are shown on the right broken down based on the time quadrant (pairwise Student’s *t* tests with Bonferroni correction: ∗∗p<0.01,∗∗∗p<0.001). (B) PDR model weights for each time quadrant. No interaction weight was found for the model trained on PDR separately for any time quadrant. The top and bottom rows are the Music and Nature experiments, respectively. The shaded area shows the SEM.

Examining patterns of feature interaction over time reveals clear changes as the auditory sequence unfolds. Analysis of the behavioral model’s learned weights reveals clear time-dependent changes in feature integration. To examine how these interactions evolve across the auditory sequence, separate behavioral models are trained for each time quadrant based on when the deviant occurred. As shown in Figure 6A, cross-feature interactions are absent in the first quadrant but emerge and strengthen in subsequent quadrants, indicating that feature integration develops as the auditory context stabilizes. Model performance measured using AUROC increases over time, yielding a lower performance in the first quadrant (Music experiment: one-way ANOVA: F=45.29,p<2.2e−16; Nature experiment: one-way ANOVA: F=35.53,p<2.2e−16). This trend parallels the behavioral responses, which reveal worse performance in the first quadrant before accuracy gradually increasing (Music experiment: one-way ANOVA: F=15.67,p=7.62e−10; Nature experiment: one-way ANOVA: F=75.44,p<2.2e−16). Results of the post hoc tests are shown in Table 2. These results suggest that the cross-feature interactions develop over time as participants accumulate information about the ongoing auditory context and gradually refine their decision-making strategies to incorporate multiple acoustic dimensions more effectively.

**Table 2:** Results of post hoc one-way ANOVA analyses comparing the AUROC of behavioral models and participants’ accuracy across four TQs

TQs	Music experiment Models’ AUROC (*p* value)	Nature experiment Models’ AUROC (*p* value)	Music experiment Participants’ accuracy (*p* value)	Nature experiment Participants’ accuracy (*p* value)
**TQ1, TQ2**	2.88e−11	2.44e−11	9.74e−7	5.11e−11
**TQ1, TQ3**	<2.2e−16	<2.2e−16	1.69e−9	<2.2e−16
**TQ1, TQ4**	<2.2e−16	5.66e−16	2.41e−4	<2.2e−16
TQ2, TQ3	0.26	0.25	1	1.11e−6
TQ2, TQ4	8.98e−4	0.83	1	8.61e−10
TQ3, TQ4	0.44	1	0.14	1

TQ, time quadrant. Bolded values indicate statistical significance (*p* < 0.05).

In contrast, the PDR model showed no evidence of time-dependent change in feature interactions (Figure 6B), with no observed cross-feature interactions across all quadrants for both Music and Nature experiments. We do note variations in self-feature weights (circle size), suggesting changes in the relative contribution of individual acoustic dimensions over time and possibly reflecting local adjustment to momentary differences in individual features. The lack of feature-interaction suggests that the physiological response shows limited evidence of changing feature interactions throughout the trial.

## Discussion

The present study set out to examine how auditory deviance is reflected in behavioral responses and autonomic arousal when multiple acoustic dimensions vary simultaneously. By embedding controlled deviant events within musical and naturalistic birdsong contexts and modeling the resulting responses within a common predictive coding framework, we compare two response modalities associated with deviance detection. This integrated approach provides a comparison of how complex acoustic inputs relate to physiological responses and perceptual judgments.

Two main observations emerge. First, behavioral detection depends on the interaction of pitch, loudness, and timbre, confirming that listeners integrate information across feature channels to build a multidimensional representation of “normality” against which violations are judged. These cross-feature interactions strengthen as the auditory scene unfolded, consistent with predictive-coding accounts in which priors are refined over time.^14^^,^^15^ Second, the PDR is well explained by feature-specific deviations in individual acoustic dimensions: loudness and pitch produce robust dilations, whereas timbre—despite being perceptually deviant in behavior—elicits only a muted response. This dissociation indicates that the two response modalities are differentially sensitive to stimulus features and suggests that the LC-norepinephrine (LC-NE) system, the primary driver of the transient phasic pupil changes,^16^^,^^17^ has been associated with sensitivity to certain types of acoustic change, such as intensity onsets and frequency glides.^18^ Complex spectral patterns that define timbre may contribute more strongly to perceptual judgments without eliciting equally strong pupil responses, potentially reflecting differences in how these features are represented behaviorally.^19^ This functional dissociation between behavior and PDR can be interpreted as reflecting different aspects of a shared underlying processing stream. Importantly, our results do not directly establish that PDR reflects an earlier or lower-level processing stage, but rather demonstrate differences in how each response modality relates to the modeled acoustic features.

The dissociation between behavioral response and PDRs also may have an evolutionary explanation. Abrupt, high-energy changes in loudness and sharp variations in pitch are primary acoustic cues for threat and urgency. Research has shown that human screams, for example, possess unique acoustic properties, such as rapid amplitude modulation or “roughness,” that are distinct from other vocalizations.^20^ These specific acoustic features have been shown to strongly engage neural systems associated with threat processing, including the amygdala, hence supporting rapid and prioritized responses to potential danger. In contrast, timbre provides more nuanced information about a sound’s identity, which is vital but perhaps less urgent than the initial detection of a threat. In this view, PDR does not reflect the full perceptual richness of auditory processing but rather indexes a subset of stimulus properties that are particularly effective in engaging arousal systems.

Expertise further modulates performance in the Nature and Music experiments. Participants are more accurate with musical than birdsong deviants, suggesting that long-term exposure and perceptual familiarity shape the internal predictive models that guide auditory deviance. The human auditory system continuously builds internal statistical models based on the regularities of its acoustic environment.^21^ Given the ubiquity of tonal music in many cultures, most listeners have implicitly acquired a sophisticated predictive model of its melodic and harmonic structures. This extensive, albeit often passive, training facilitates the efficient detection of events that violate learned expectations, a phenomenon well-documented in studies of music cognition.^22^ Conversely, the complex acoustic structures of birdsong are likely less familiar to the average listener, resulting in a less-refined internal model and, consequently, poorer deviant detection performance.

An alternative, although not mutually exclusive, explanation lies in the fundamental acoustic properties of the stimuli. The birdsong calls used in our experiment were characterized by higher average spectral frequencies than the musical tones. It is a well-established principle of psychoacoustics that human frequency resolution declines at higher frequencies, meaning a larger objective change is required to be perceived as different.^23^ Therefore, the observed performance gap may be partially attributable to the inherent difficulty of discriminating changes in the higher-frequency range of the birdsong stimuli, independent of listener familiarity. It is plausible that these two factors—acoustic constraints and long-term learning—interact, as the brain’s ability to build robust predictive models may be constrained by these fundamental limits of sensory processing. Future studies could directly test the expertise hypothesis by using computational models of statistical learning, pre-training them on corpora with varying proportions of music and nature sounds to simulate different listening histories and determine if they replicate the human performance asymmetry.

Methodologically, this study demonstrates the value of combining computational modeling with behavioral and physiological measures to probe auditory processes in response to deviant sounds. By linking complex acoustic inputs to both perceptual judgments and autonomic responses, the approach reveals how predictive computations shape distinct aspects of perception. While prior computational models have successfully predicted auditory salience from acoustic features, the D-REX model offers a more flexible framework compared to the Kalman filter used in a previous study by Kaya and Elhilali.^13^ Its architecture, which tracks statistical regularities in the input features, allowed us to generate an estimate of stimulus surprisal and quantitatively test how this signal relates to different response modalities. The stimuli in this study contained controlled deviations, permitting examination of how different feature combinations contribute to perceived and physiological deviance. Importantly, previous work using the same musical paradigm has demonstrated that these acoustic deviants evoke neural signatures of auditory salience even when unattended, validating their use as stimuli for probing salience-related processing.^24^ Using these stimuli and this framework, one can move beyond descriptive analysis to investigate causal hypothesis regarding the computations underlying salience detection.

The modeling framework presented here offers several promising avenues for future investigation. First, the model’s parameters could be fitted to individual participant data to capture their listening strategies, which are known to vary significantly across the population, particularly in complex listening tasks.^25^ Second, the framework could be extended to explore the role of higher-order statistics, testing whether the brain’s internal models represent more complex aspects of acoustic features.^26^

In conclusion, this study sought to determine whether the PDR reflects the complex, multi-feature integration seen in conscious auditory perception or acts as a direct marker of sensory deviance. By employing a predictive coding framework (D-REX) to model both physiological and behavioral data, we uncovered a fundamental dissociation. While behavioral judgments were sensitive to the interactive statistics of our acoustic scenes, PDR primarily indexed a generalized “prediction error,” largely independent of these complexities and interactions. These findings indicate that PDR and behavioral responses reflect different facets of how auditory deviance is encoded. PDR appears to provide a readout of arousal-related responses to deviant stimulus changes, whereas behavioral responses capture additional, more integrative aspects of auditory processing. By linking stimulus features to distinct response modalities through a computational model, this work provides a framework for exploring how different mechanisms contribute to human auditory perception.

### Limitations of the study

Despite its contributions, this study has several limitations that point to important directions for future research. First, the current computational framework does not fully capture the full range of time scales, specifically longer time scales, which may be more appropriate for explaining PDRs. Although we implemented several measures in the current setup to account for structural differences between the model’s responses and PDRs, exploring model modifications that capture PDR dynamics more accurately could provide valuable insights. Second, although D-REX provides a framework for quantifying different response types, fundamental differences between behavioral and PDR measures make direct comparisons challenging. Behavioral responses are discrete and trial level, whereas PDRs are continuous and typically averaged over conditions or subjects. Conducting a cross-modality analysis to directly compare these responses would be a valuable next step, but it would require a larger dataset with more trials and participants. Third, while D-REX provides a principled way to link feature-level surprisal to observed responses, direct validation against direct measures of neural activity is required. Integrating this approach with high-temporal-resolution methods like magneto- or electroencephalography would be a powerful next step. The surprisal signal computed by D-REX, for instance, provides a quantitative hypothesis that can be directly mapped onto neural signatures of predictive coding, such as the mismatch negativity event-related potential.^27^ Together, these complementary approaches provide a comprehensive framework linking stimulus acoustics to the underlying neural computations, and ultimately to their expression as both physiological arousal and conscious behavioral responses. Finally, the current study used group-level models, which may obscure individual variability in salience processing. Future work could fit model parameters at the individual level to uncover how expertise, attention, or learning history shape predictive coding in different listeners.

## Resource availability

### Lead contact

Further information and requests for resources should be directed to and will be fulfilled by the lead contact, Mounya Elhilali (mounya@jhu.edu).

### Materials availability

This study did not generate new unique reagents.

### Data and code availability

• Data: Behavior and pupillometry datasets for this study have been deposited in the Open Science Framework (OSF) and are publicly available at OSF: https://doi.org/10.17605/OSF.IO/FZ2KJ.
• Code: Example scripts for testing the model introduced in this study are publicly available on OSF: https://doi.org/10.17605/OSF.IO/FZ2KJ.
• Additional information: Any additional information required to reanalyze the data reported in this paper is available from the lead contact upon request.

## Acknowledgments

This work was supported in part by ONR N00014-23-1-2050 and NSF 2444353-01.

## Author contributions

H.-I.L. conducted the experiments, processed pupillometry data, and analyzed behavioral data. N.F. and M.E. designed the computational framework. The first draft was written by N.F., and all authors contributed to writing and editing the manuscript.

## Declaration of interests

The authors declare no competing interests.

## STAR★Methods

### Key resources table

**Table:** 

REAGENT or RESOURCE	SOURCE	IDENTIFIER
**Software and algorithms**		

MATLAB R2018b and R2022b	MathWorks https://www.mathworks.com/	RRID: SCR_001622
Psychophysics Toolbox Version 3	http://psychtoolbox.org/	RRID: SCR_002881
Custom MATLAB Code	This paper; Open Science Framework (OSF)	OSF: https://doi.org/10.17605/OSF.IO/FZ2KJ

**Deposited data**		

Behavioral and Pupillometry Data	This paper; OSF	OSF: https://doi.org/10.17605/OSF.IO/FZ2KJ

**Other**		

SR Research EyeLink Eye Trackers (Eyelink 1000 Desktop Mount)	SR Research Ltd. https://www.sr-research.com	RRID: SCR_009602

### Experimental model and study participant details

#### Participants

Thirty-one adults (24 women, age range 20-48 years, median age 40 years, see Table S1 for more detail.) participated in the current study consisting of two experiments: 16 participants in the Music Experiment and 15 in the Nature Experiment. All participants reported normal or corrected-to-normal vision and normal hearing acuity. Sample sizes were chosen based on our previous studies with comparable pupillometry measurements.^5^^,^^28^^,^^29^ The current study was approved by the NTT Communication Science Laboratories ethics committee (H28-006). All participants gave written informed consent before the experiment and received payment for their participation.

#### Apparatus and stimuli

Visual and auditory stimuli were generated by MATLAB (MathWorks, Inc.) with PsychToolbox.^30^ Visual stimuli were presented on an 18.1-inch monitor. A small dark gray dot (0.25 ° × 0.25 °, 0.33 cd/m^2^) served as the fixation point and was presented at the center of the monitor against a light gray background (27.0 cd/m^2^). Auditory stimuli were presented through two loudspeakers at a comfortable listening level, self-adjusted by each participant. They were either brief musical clips (5 seconds in the Music Experiment) or birds’ singing calls (6 seconds in the Nature Experiment), consisting of temporally overlapping sound tokens, identical to those used in the study by Kaya & Elhilali.^13^ The auditory streams contained both regular and deviant acoustic tokens. The deviant token was designed to stand out from the background sounds due to a noticeable deviation in acoustic features, including loudness, pitch, or timbre. In the Music experiment, the deviant token was a note at 2 or 6 dB (loudness) and 2 or 6 semitones (pitch) higher than the background. The timbre of the deviant token was either the same as or different from the background, depending on whether a pianoforte or an acoustic guitar was assigned as the foreground and background instrument. In the Nature Experiment, the foreground note was at 2 or 8 dB (loudness) and 0 or 3 semitones (pitch) higher than the background. The songs were sung by Common Yellowthroat or MacGillivray’s Warbler. To minimize predictability, the deviant token appeared at a randomly assigned time within one of the four equal-length quadrants of each sound clip in half of the trials. In the remaining half, which were the control trials, only the background scene was presented with no deviant tokens included. The sound tokens in the Music experiment were 1.2s long, and their durations varied between 1.3s-1.5s in the Nature experiment. An example of stimuli containing deviant tokens is shown in Figure 1.

Participants’ eye movements and pupil responses were recorded by an infrared-based eye-tracker camera (Eyelink 1000 Desktop Mount, SR Research Ltd.). Before each experimental session, participants went through the 5-point Eyelink calibration procedure to calibrate and validate their eye movement data. They were instructed to fixate the fixation point after the calibration and throughout the experiment. To ensure the pupil responses returned to the baseline, we inserted a 5-second interval between the trials. Each experimental session contained 128 trials and took approximately 25 minutes.

#### Procedure

All participants were given written and oral explanations of the experiments and the pupil response recordings. They sat in front of the monitor at a viewing distance of 80 cm in a dimly lit chamber, with their heads fixed on a chinrest. Their pupil diameters were measured while they performed the auditory deviant token detection task. In each trial, after the sound presentation, they were asked to press the “space” key on the keyboard within 2 seconds if they detected the deviant token. They did not need to press any key if they did not notice the deviant token. They took several practice trials until they were familiar with the task. During the practice, feedback of “Correct!” or “Wrong!” was shown on the screen after their response (or 2 seconds after the sound presentation if there was no key pressing). No feedback was given in the formal experiment. Each participant performed two formal experimental sessions, with a 20-mintues break between the sessions. By analyzing both the pupillary responses and participants’ behavioral reports, the experiment aimed to examine the relationship between physiological indicators (pupil dilation response, PDR) and subjective detection of deviant acoustic events.

### Method details

#### Analysis of behavioral and physiological data

##### Participants’ behavioral response analysis

The same procedure as in Kaya and Elhilali^13^ is followed to calculate the $d^{\prime }$ metric parameterized by the type of deviant tokens (2 loudness level × 2 pitch difference × 2 background timbre × 2 foreground timbre). Hit rate is the average of the correct detections in each factorial condition. False alarm rate is the average of the false detections of the control trials, constant for each participant. For both hit and false alarm rates, values of 0 and 1 are adjusted to 0.01 and 0.99, respectively, to avoid infinite values of $d^{\prime }\text{.}$ A four-way repeated-measure ANOVA is performed to evaluate the effect of different acoustic dimensions (Table 1).

##### Pupil response analysis

Since eye movements and pupil responses are consensual, only the data from the right eye are used. During the sound presentation period, blinks accounted for 32.1% of data points in the Music Experiment and 28.3% in the Nature Experiment. Gaze fixations deviated from the fixation point by more than two visual degrees are discarded (7.8% and 15.5% in the Music and Nature Experiments, respectively). The sampling frequency for the pupil responses is 1000 Hz and data is not low pass filtered. The missing pupil-diameter data during blinks or gaze deviation are interpolated by using shape-preserving piecewise cubic interpolation. To compare the pupil responses across participants and conditions, pupil diameter data are normalized by z-transform using all the data recorded in each experimental session and then baseline-corrected trial-by-trial by subtracting the mean of the data during the 1-second period before the sound presentation. Only the correctly responded trials (i.e., hit in the deviant token presented trials and correct rejection in the control trials) are included in the pupil analysis.

#### Computational model

##### Computational features

The computational model examines three acoustic attributes of the stimuli: Loudness, Pitch and Timbre. Each attribute is represented along multiple acoustic dimensions. First, the audio signal is downsampled from its original 44.1 kHz sampling rate to 16 kHz. Loudness is extracted using the envelope of the signal, derived from the magnitude of the Hilbert transform which is Butterworth filtered with $w_{c}=40$ and n=6. Both Pitch and Timbre are estimated from an auditory spectrogram that maps the time waveform onto a time-frequency channel following the model introduced by Chi et al.^11^ Estimates of fundamental frequency are extracted using a harmonicity analysis of the auditory spectrogram,^12^ which is computed using a bank of 128 bandpass filters which are equally spaced along a logarithmic frequency axis and their center frequencies span a range of 5.3 octaves (180-7246 Hz for a 16 kHz sampling frequency). First, the pitch estimates are smoothed by taking their log, as it helps with reducing large drops or rises in the estimates. Then, they are further smoothed using a median filter with a window length of 5 and the first derivative is used to remove sudden large drops or rises. Points where the derivative deviates from its mean by more than two standard deviations are replaced with the preceding pitch estimation value. In addition, the spectral responses from low-frequency channels ($f_{c}<1120$ Hz) of the spectrogram are averaged to provide a supplemental estimate of spectral energy of resolved harmonics hence complementing information derived directly from the harmonicity structure of each spectral slice. Timbre is represented using different dimensions. The average spectral energy in the high-frequency channels of the auditory spectrogram ($f_{c}>1120$ Hz) is quantified as a function of time to capture resonances in the sound. In addition, the spectrogram is further mapped onto temporal and spectral modulations using a wavelet decomposition following the cortical model in Chi et al.^11^ Filters with characteristic frequencies logarithmically distributed between $2^{-4}$ and $2^{4}$ cycles/octave are applied to the spectral slices of the audio spectrogram to extract the scale information. The resulting cortical representation of the audio is averaged over frequency channels and different scales/rates to compute one representation of scale/rate over time.

Altogether, this process produces six acoustic features that vary over time for each sound, which are then analyzed through a predictive coding framework, described next.

##### Surprisals

Unlike the Kalman filter approach employed in,^13^ the current study uses the Dynamic Regularity Extraction (D-REX) model as predictive coding framework,^10^ which offers more flexibility to represent uncertainty and context beliefs without assuming linear dynamics or Gaussian noise. D-REX is based on Bayesian Inference that builds a predictive distribution of any acoustic attributes as it evolves over time and updates the sufficient statistics for its underlying probability distribution (e.g., Gaussian). The model keeps track of multiple context hypotheses and assigns a belief value to each of the context-specific predictions. Then, by calculating a weighted sum over context beliefs, it can produce a surprisal value (in bits) for each observation. The surprisal of the observation $x_{t}$ shows the mismatch between the observation and its predictive probability and how strong the context beliefs are for that input. Therefore, if there is a change in the underlying distribution of the input feature at time t, the surprisal for $x_{t}$ would be high, indicating that $x_{t}$ deviates from the model’s expected distribution.

In the present study, the six features (envelope, pitch, low-frequency spectrogram, high-frequency spectrogram, scale and rate) are downsampled to 10Hz and given as input to the D-REX model to compute a set of surprisal values $s_{i}\left(t\right)\left(1\leq i\leq 6\right)$ for each feature. Model parameters were selected heuristically to optimize detection of deviant events while ensuring temporal smoothness. The temporal dependence parameter (set to D=1) controls the influence of past samples on the current prediction. The hazard rate (set to 0.5) reflects the assumed probability of a change in statistical context, balancing sensitivity to abrupt changes against noise robustness. Each trial’s prior distribution is initialized using statistics from its first time quadrant, enabling the model to adapt to the local acoustic environment. These parameter choices allow D-REX to dynamically capture the evolving statistical structure of complex auditory scenes and generate feature-specific surprisal signals suitable for modeling deviance detection across behavioral and physiological domains.

##### Feature integration

Expanding on the methodology introduced by Kaya & Elhilali,^13^ a logistic regression model is used to estimate the likelihood of a deviant event occurring in an auditory stream, based on time-varying surprisals of acoustic features. Surprisal, in this context, quantifies how unexpected or deviant a particular feature is at a given moment relative to its acoustic context. Ultimately, the goal is to map these feature-specific surprisals to an integrated model output α(t) that represents the estimated probability of a deviant event at each time point.

To incorporate cross-dimensional feature interactions, we extend the original surprisals estimated for each feature by augmenting the surprisal $s_{i}\left(t\right)$ with a self-weight $w_{ii}$ and interaction with other features $w_{ij}$ over a short temporal window. The boosted surprisal, $s_{i}^{\prime }\left(t\right)$, for feature i at time t is defined as:

(Equation 1) $$s_{i}^{\prime }\left(t\right)=s_{i}\left(t\right)\left(w_{ii}+\sum_{\begin{array}{c} j=1\\ j\neq i \end{array}}^{6}w_{ij}\max_{\tau \in \left[-T,T\right]}s_{j}\left(t+\tau \right)\right)$$

Here, $s_{i}\left(t\right)$ is the original surprisal of feature i derived from the D-REX model. $w_{ii}$ is the learned self-connection weight boosting the surprisal for feature i; $w_{ij}$ is the learned interaction weight between feature i and j, and T=100ms defines the temporal window within which interactions are computed. This formulation captures not only the individual contribution of a feature but also how its salience is modulated by the presence of deviant events in other dimensions within a brief temporal context. The choice of temporal window T is empirically derived but alternative values within a reasonable range did not quantitatively change the overall results.

Additional preprocessing is applied to the surprisals $s_{i}^{\prime }\left(t\right)$ to further sparsify peaks in the surprisal signal. First, the surprisal values are normalized to the [0,1] range for each feature using their minimum and maximum values across all trials. Next, a threshold is applied to zero out values below the 70th percentile, yielding a spiky temporal representation.

Two separate regression models are trained using this representation: one based on participants’ *behavioral* responses and the other using their *pupil dilation response* (PDR) as ground truth. In both cases, the input features of the regression are the augmented surprisals $s_{i}^{\prime }\left(t\right)$, and the regression output variable is y(t), defined as

(Equation 2) $$y\left(t\right)=\left\{ \begin{array}{ll} 1 & t_{0}\leq t\leq t_{0}+\Delta t\\ 0 & \text{otherwise} \end{array}\right.$$

where $t_{0}$ is the onset time of the deviant stimulus (or the maximum PDR peak within a constrained window), and Δt defines the temporal window around the deviant event used for labeling salience. Given the sound token lengths in each experiment, with the Music experiment featuring tokens of 1.2 seconds and the Nature experiment featuring tokens varying between 1.3 and 1.5 seconds, Δt was set to 1.2 seconds for the Music experiment and 1.4 seconds for the Nature experiment. Details on the choice of $t_{0}$ for the behavioral and PDR outputs are outlined in the “model training and evaluation: behavioral responses” and “model training and evaluation: pupil dilation response (PDR)” sections below. y(t) indicates the existence of a deviant in the stimuli over time. For the control trials, y(t) is equal to zero for all t. Each feature is processed separately to reduce noise, similar to Kaya and Elhilali’s method.^13^ Model training is performed by maximizing the log-likelihood function:

(Equation 3) $$L=\max_{w_{ij}}\sum_{t}\log\left(\frac{y\left(t\right)+{\left(-1\right)}^{y\left(t\right)}2^{\left(1-y\left(t\right)\right)}e^{-s_{i}^{\prime }\left(t\right)}}{1+e^{-s_{i}^{\prime }\left(t\right)}}\right)\;st.\;w_{ij}\geq 0$$

The probability of a deviant event is computed for each feature ($\alpha_{i}\left(t\right)$) using the trained weights and a sigmoid function (Equation 4). The model’s final output, α(t), is the average over $\alpha_{i}\left(t\right)$ and represents the estimated probability of a deviant event occurring at each time point. Figure 2 presents an overview of the modeling pipeline.

(Equation 4) $$\alpha_{i}\left(t\right)=\frac{2}{1+e^{-s_{i}^{\prime }\left(t\right)}}-1$$

(Equation 5) $$\alpha \left(t\right)=\frac{1}{6}\sum_{i=1}^{6}\alpha_{i}\left(t\right)$$

To compare the integration strategies of behavior and PDR models, we examine the resulting interaction weight matrices from each model. These 6×6 matrices are further reduced to 3×3 matrices by grouping acoustic dimensions into perceptual attributes: pitch is represented by combining the pitch estimate and low-frequency spectrogram; timbre includes the high-frequency spectrogram, scale, and rate; and loudness is captured by the envelope. By comparing the structure and magnitude of these reduced matrices, we assess whether behavioral and physiological responses rely on similar patterns of multidimensional feature integration.

##### Model training and evaluation: behavioral responses

To assess how well behavioral responses relate to auditory deviance, we train a regression model using participants’ detection responses. The model is trained on behavioral responses by converting the binary behavioral response to a step signal, y(t) (Equation 2). To do so, trials are categorized based on whether a deviant token is detected. In trials with correct detections (true positives), the target label $t_{0}$ (Equation 2) is aligned to the ground-truth onset time of the deviant. In false alarms, where participants respond despite no deviant being present, $t_{0}$ is assigned to the peak of the summed surprisal across all features. Misses and correct rejections are both considered as control trials for training, meaning y(t) in Equation 3 is set to zero for all t.

A leave-one-subject-out cross-validation strategy is utilized. For each fold, the model is trained on all data from all participants except one, then evaluated performance on the held-out participant. The learned weight matrices are averaged across training subjects and used to generate a deviance probability time series α(t) for each trial in the test subject. Model performance is quantified by constructing receiver operating characteristic (ROC) curves, by comparing α(t) against a threshold varying between 0 and 1, and computing the area under the ROC (AUROC). Two baseline models are used for comparison: (1) a uniform interaction model that assigns equal weights to all features (i.e., identity W matrix with no learned feature interactions), and (2) a random baseline where deviant onset labels are shuffled across trials during training. Each random model is trained ten times per subject, and average performance is used for evaluation.

##### Model training and evaluation: pupil dilation response (PDR)

Given the variability of trial-level PDR, model training is performed on condition-averaged traces. Specifically, for each of the 32 experimental conditions (2 pitch × 2 loudness × 2 timbre × 4 time quadrants), average PDR responses are computed for each participant across all non-control trials. The analysis is also repeated with trials excluded in which deviants are not correctly detected or rejected by the participants. As the results are not meaningfully affected by this exclusion, only the findings based on the full set of trials are reported. To generate a balanced training set, control trials are randomly grouped into clusters of four to match the number of conditions in the deviant trials. Each grouped control set yields a corresponding averaged PDR trace. For each averaged deviant trial, the target time $t_{0}$ is defined as the peak of the PDR within a one-second window following the ground-truth deviant onset, accounting for physiological delay. This approach differs from the behavioral model training, as PDRs are inherently noisy continuous-time signals. Accordingly, we adopt a distinct procedure to convert them into the step signal y(t) (Equation 2).

As with behavioral data, model training follows a leave-data-out approach. For each fold, the model is trained on averaged PDR data from eight participants. Predictions α(t) are then averaged across conditions to yield a condition-level estimate of deviance. Subsequently, the model’s output generated from a specific set of training weights is evaluated by comparing it against the averaged PDR of the eight excluded participants. Performance is assessed via maximum cross-correlation between model output and empirical PDR across the 32 conditions. Cross-correlation is computed within a three-second lag window and normalized using zero-lag autocorrelations. A random baseline model is constructed by training on shuffled deviant onset times, repeated five times, and the results are averaged for comparison. To align the format of empirical and modeled data, PDR traces are binarized using a 90% peak threshold to yield temporally discrete responses.

##### Time-dependent models by quadrants

To examine how interaction weights and model performance evolve with the timing of the deviant token, we train four separate models (one for each time quadrant) on both behavioral responses and PDR. The training and testing procedures follow the same approach as described above.

To ensure balanced train and test sets with equal numbers of control and non-control trials in each quadrant, a bootstrapping strategy is implemented. Specifically, for each model, a quarter of the control trials are randomly selected, making sure that all control trials are utilized across one full training pass of the four models. This process is repeated ten times, each with a new combination of control trials. As a result, the performance and weights represent the average across participants and bootstrap iterations.

### Quantification and statistical analysis

The behavioral $d^{\prime }s$ are subjected to a four-way repeated-measure ANOVA with Loudness, Pitch, Timbre-background, and Timbre-foreground as within-subject factors. Another repeated-measure ANOVA, including deviant token presentation time, is performed with the acoustic features (Loudness, Pitch, Timbre), change differences (small, large), and times (TQ1, TQ2, TQ3, TQ4) as within-subject factors and experiments (Music, Nature) as the between-subject factor.

To test the significance of pupil responses, linear mixed-effects (LME) analyses are performed at each 1-msec sampling point, with the deviant token presented or not as the fixed effect, participant as the random effect, and pupil diameter as the dependent variable. To examine how different acoustic features (Loudness, Pitch, and Timbre) affect PDR at each time quadrant, separate LME analyses are performed with the acoustic feature difference (e.g., 2 and 6 dB in the Music Experiment for Loudness) as the fixed effect, participant as the random effect, and pupil diameter as the dependent variable. The LME analyses are computed by using the *lem4* package in R.^31^ Following the same criterion in Mathot et al.,^32^ the significant clusters were defined as a *t* value >2 with at least 200 consecutive samples.

To compare the computational models, a one-way ANOVA is used to assess whether the behavioral, uniform, and random models exhibit significantly different performance in terms of AUROC. Pairwise Student’s t-tests are then conducted to identify which model pairs differ significantly. The p-values from these comparisons are adjusted using the Bonferroni correction. To compare the correlation of the PDR model and the random model with the PDR, a Student’s t-test is performed to determine significance. Additionally, a One-Way ANOVA and t-test post-hoc with Bonferroni correction are used to compare the AUROC of the behavioral model with subjects’ accuracy across different time quadrants.

### Additional resources

Further information and requests for resources should be directed to and will be fulfilled by the lead contact, Mounya Elhilali (mounya@jhu.edu).
