# Supplementary material for: Dissociation in cross-feature integration between behavioral and pupil dilation responses in auditory deviant detection
Source: iScience. 2026 May 20;29(6):116013. doi: 10.1016/j.isci.2026.116013 (PMC13214528; doi:10.1016/j.isci.2026.116013)
Supplement: Document S1. Figure S1 and Table S1 [file mmc1.pdf]

iScience, Volume *29*

Supplemental information

Dissociation in cross-feature integration between behavioral and pupil dilation responses in auditory deviant detection

Nahaleh Fatemi, Hsin-I Liao, and Mounya Elhilali

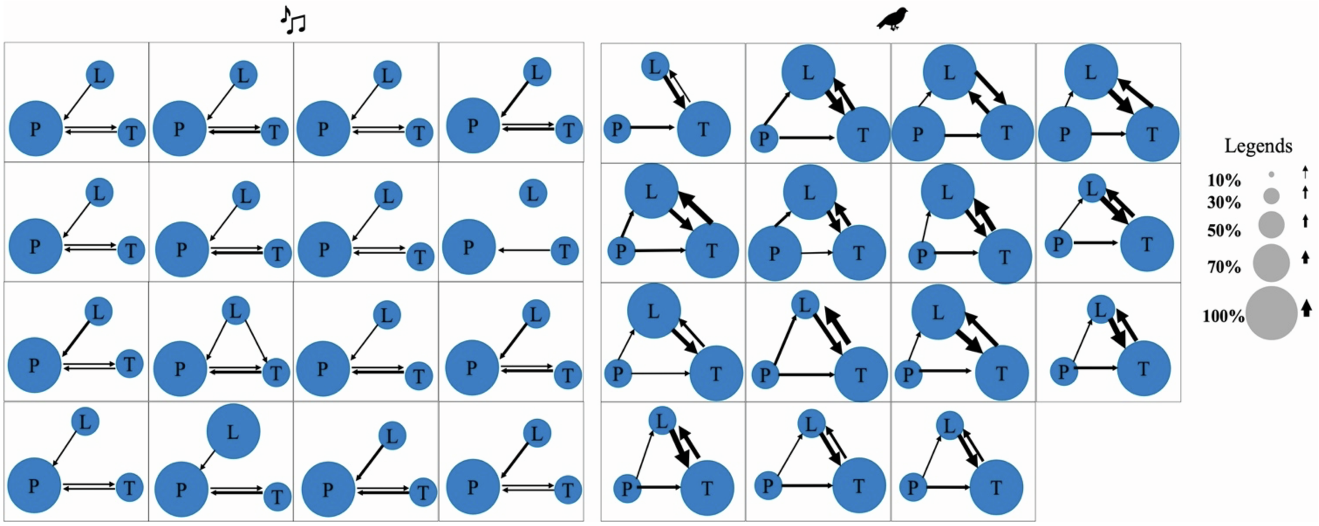

Figure S1: Behavioral Weights Shown for Each Participant, related to Figure 4C. Left: music, right: nature experiment. Figures show the feature interactions from the model trained on behavioral responses for each subject. The weights are consistent across participants for the two experiments. Legends are shown on the right.

Table S1: Participants' Clinical and Demographic Details.

Subject ID	Age	Gender	Race	Ethnicity	Neurological disorder
E1-1	31	F	Asian	Japanese	None
E1-2	34	F	Asian	Japanese	None
E1-3	38	F	Asian	Japanese	None
E1-4	37	F	Asian	Japanese	None
E1-5	37	F	Asian	Japanese	None
E1-6	24	M	Asian	Japanese	None
E1-7	40	F	Asian	Japanese	None
E1-8	40	F	Asian	Japanese	None
E1-9	47	F	Asian	Japanese	None
E1-10	42	F	Asian	Japanese	None
E1-11	44	F	Asian	Japanese	None
E1-12	40	M	Asian	Japanese	None
E1-13	42	F	Asian	Japanese	None
E1-14	43	F	Asian	Japanese	None
E1-15	46	M	Asian	Japanese	None
E1-16	48	M	Asian	Japanese	None
E2-1	41	F	Asian	Japanese	None
E2-2	21	M	Asian	Japanese	None
E2-3	45	F	Asian	Japanese	None
E2-4	39	F	Asian	Japanese	None
E2-5	20	M	Asian	Japanese	None
E2-6	41	F	Asian	Japanese	None
E2-7	38	F	Asian	Japanese	None
E2-8	42	F	Asian	Japanese	None
E2-9	32	F	Asian	Japanese	None
E2-10	43	F	Asian	Japanese	None
E2-11	20	M	Asian	Japanese	None
E2-12	47	F	Asian	Japanese	None
E2-13	39	F	Asian	Japanese	None
E2-14	35	F	Asian	Japanese	None
E2-15	38	F	Asian	Japanese	None
